# Heat Tolerance of Siberian Husky Dogs Living in Brazil: A Case Study on the Perceptions and Attitudes of Their Owners

**DOI:** 10.3390/ani13172774

**Published:** 2023-08-31

**Authors:** Tarsys Noan Silva Veríssimo, Edilson Paes Saraiva, Aline Cristina Sant’Anna, Bruna Agy Loureiro, Pavlos Vinicius do Nascimento, Luiz Arthur dos Anjos Lima, Maria Isabelly Leite Maia, Larissa Kellen da Cunha Morais, Severino Guilherme Caetano Gonçalves dos Santos, Eduardo Henrique Santos de Lima, Vinícius de França Carvalho Fonseca

**Affiliations:** 1 Research Group in Bioclimatology, Ethology and Animal Welfare (BioEt), Department of Animal Science, Federal University of Paraíba, Areia 58397-000, Brazil; verissimotns@hotmail.com (T.N.S.V.); edilson@cca.ufpb.br (E.P.S.); pavlosvinicius@hotmail.com (P.V.d.N.); luisarthur_@hotmail.com (L.A.d.A.L.); isabelymaia12@gmail.com (M.I.L.M.); larissakcm@gmail.com (L.K.d.C.M.); ehslmvz@gmail.com (E.H.S.d.L.); vinicius_fonseca86@hotmail.com (V.d.F.C.F.); 2 Nucleus of Studies in Ethology and Animal Welfare, Departament of Zoology, Federal University of Juiz de Fora, Juiz de Fora 36036-900, Brazil; 3 Veterinarian Medicine and Animal Science School, Federal University of Bahia, Salvador 40170-110, Brazil; brunaagy@yahoo.com.br; 4 Department of Animal Production, National Institute of Semiarid—INSA, Campina Grande 58429-970, Brazil; guilhermeufpb@gmail.com

**Keywords:** heat, hyperthermia, owners, pets, solar radiation, stress, temperature, welfare

## Abstract

**Simple Summary:**

The high radiant heat load of tropical regions imposes challenges on the welfare of imported temperate dog breeds, especially when owners’ awareness does not result in willingness to change their attitudes towards the care of their dogs, e.g., by choosing more thermally comfortable times to walk with them. Based on an online questionnaire answered by Siberian Husky dogs’ owners, we found that respondents mentioned several heat defense behaviors expressed by their dogs, perceived them as low-heat-tolerant animals and were more likely to walk with dogs during times of less solar input. However, the availability of time was reported by owners as the main reason for taking their dogs for a walk during times of high solar input. In conclusion, the owners’ perception that Siberian Huskies living in Brazil are heat-sensitive dogs was likely to reflect positive attitudes via avoidance of exposing them to extreme hot conditions during daily walks.

**Abstract:**

The management of the thermal environment to which dogs are exposed should be included in strategies to improve their welfare. An online questionnaire was administered to 624 owners of Siberian Husky dogs residing in Brazil, with the objective of assessing their perceptions regarding their dogs’ capacity to adapt to heat, and its association with the owners’ routine care. Owners who believed that dogs are low-heat-tolerant animals were more likely to report heat response behaviors from their dogs. Overall, owners reported walk with their dogs during early morning, late afternoon and nighttime. They also reported solar radiation as the primary criteria for determining the time to walk with their dogs. However, owners who reported walking with their dogs at noon mentioned time availability as their primary criteria. In conclusion, owners perceive Siberian Husky dogs living in Brazil as being poorly adapted to heat, and this perception appeared to influence their positive attitudes towards protecting their dogs from heat stress by choosing to walk them during times with less solar exposure. However, the lack of time for owners to walk with their dogs during cooler periods can still be a risk factor in exposing the animals to extreme hot conditions.

## 1. Introduction

Dogs are increasingly occupying spaces in human homes, being considered by many as family members [1]. The growth of the pet market has provided, in addition to various niches of products and services, an increased interest in research that has potential to improve dogs’ welfare, through evaluations of the benefits of the interaction between dogs and humans [2,3] and sources of environmental enrichment [4,5,6]. For instance, for dogs who live in limited spaces, daily walks have been reported to be a good strategy to improve their welfare [7,8]. However, while walks are strongly associated with the improvement of the dogs’ quality of life, the thermal conditions imposed on them during walks can potentially compromise their welfare [9,10].

When dogs are exposed to high levels of radiant heat load, they may face challenges in offsetting the amount of heat produced and gained from the surrounding environment. Under such circumstances, body heat becomes stored, which increases the risk of hyperthermia, heatstroke and death [11,12]. Indeed, several cases of heatstroke in dogs have been reported where the principal risk factor is exposure to extreme hot weather conditions [13]. In tropical areas as in Brazil, depending on the time of day, dogs can experience as much as 1200 W m^−^^2^ of impinging solar radiation and up to 40 °C of radiant temperature during walks [14]. Furthermore, due to the rapid advances of climate change, extreme weather events like heat waves are predicted to become more frequent and intense [15]. As a result, the susceptibility of dogs to face heatstroke is expected to increase. This prospect is likely to be even more unfavorable for breeds naturally selected in cold climates but kept in hot regions.

**Figure 1:**
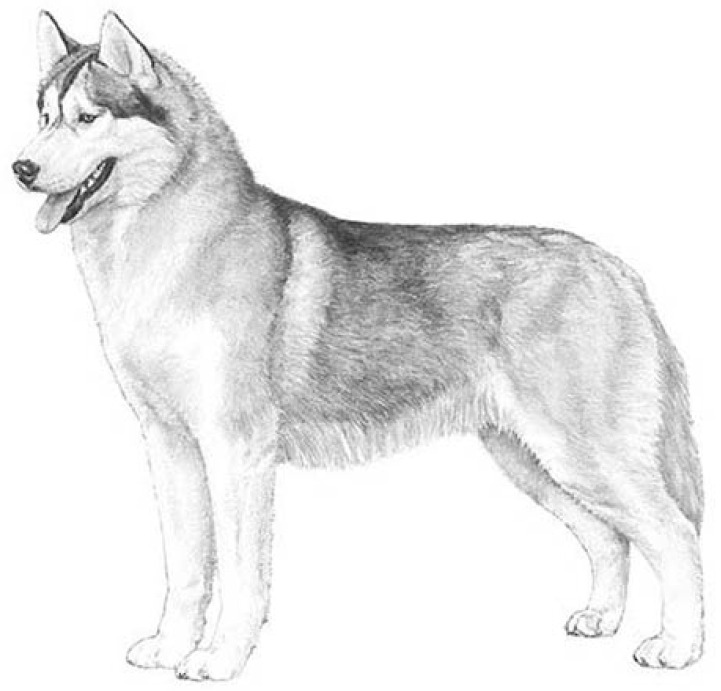
Image of a breed standard Siberian Husky specimen. Source: Brazilian Society of Cinophilia (www.sobraci.com.br, accessed on 15 August 2023).

Among the breeds originating from cold climates living in Brazil is the Siberian Husky (Figure 1), a dog from Siberia, artificially selected for pulling sleds in the snow. This breed was introduced to the United States in 1908 and recognized in 1930 by the American Kennel Club [16]. As far as we are aware, there are no reports in the literature of when and why this breed was imported to Brazil. It is likely that its beauty gained notoriety among dog lovers, and it was disseminated as a companion dog in Brazil. The high occurrence of Siberian Husky dogs and other cold-adapted breeds in hot regions raise questions about owners’ perceptions of the heat tolerance of their dogs, and the association with some of their attitudes; for example, if they choose more thermally comfortable times for daily walks. To answer these questions, an online questionnaire was sent to owners of Siberian Husky dogs living in Brazil in order to understand their perception of the heat adaptation of dogs, and the association with caring for their dogs on a routine daily basis. This study is an initial step to elaborate feasible strategies to ameliorate the impact of heat stress on dogs’ welfare.

## 2. Materials and Methods

### 2.1. Questionnaire Targeting and Structure

An online questionnaire was sent to owners of Siberian Husky dogs living in Brazil. Following the Brazilian Standards of Ethics in Scientific Research Involving Human Beings (Resolution nº 510/2016 of the National Health Council), the research was characterized as an opinion survey, with no identified respondents, and anonymity and confidentiality were guaranteed. A total of 624 questionnaires were answered. It was explained to each respondent that their participation would not imply any type of financial remuneration and that they could withdraw from answering the questionnaire at any time.

The questionnaire consisted of 15 questions (Table 1) divided into two categories: (1) owners’ knowledge about the breed and care of their dogs; and (2) the heat-related thermoregulatory behaviors expressed by their dogs. Respondents had access to the questionnaire through links on some social networks (Facebook ™, Instagram ™ and WhatsApp ™). The survey tool used was ‘Google Forms’ (Google™), which is a free online platform. Respondents could only participate if they met the criteria of owning at least one purebred Siberian Husky. To ensure that respondents owned purebred dogs, the online form was sent to many accredited breeders and made available on Siberian Husky owner groups. Data collection took place from 19 June 2019 to 22 February 2020.

### 2.2. Statistical Analysis

The questionnaire measured continuous, ordinal and nominal data (multiple-choice questions). In this way, categorical principal component analyses (PCA) were used. Categorical PCA is an analysis similar to PCA for continuous data related to objectives, results and interpretations, although it is more appropriate for different types of variable scales; that is, qualitative variables (nominal and ordinal) and quantitative variables [17]. A chi-square test in a contingency table (or Fisher’s exact test in 2 × 2 tables) was used to estimate associations between the owner’s perception about the heat adaptation of their dogs, daily routine care and observed thermoregulatory behavior of dogs. All statistical analyses were performed using the SPSS Statistics software, version 21.

## 3. Results

**Figure 2:**
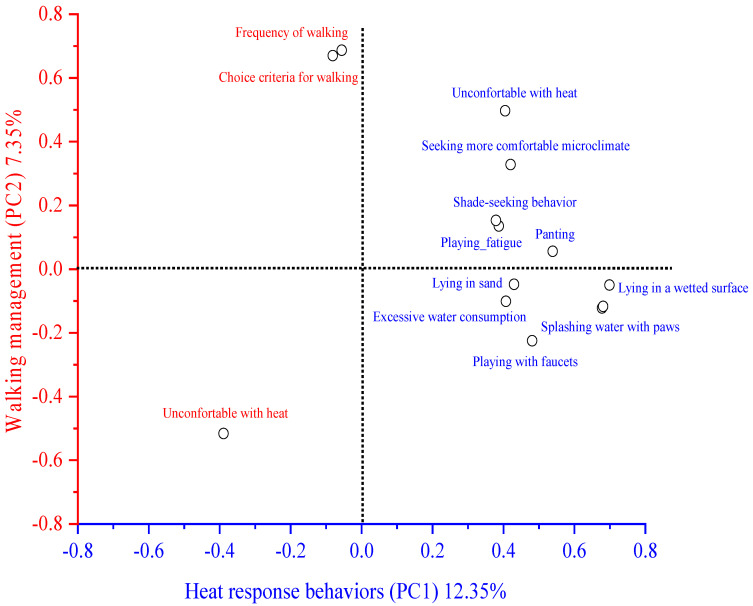
Variables in the two principal components.

Figure 2 shows that the variables that most contributed to the two principal components were related to (1) walking management (e.g., frequency of walks and criteria for choosing the time to walk with dogs) and (2) heat-related behaviors expressed by dogs. There were no associations between the perceptions of owners about the heat sensitivity of their dogs with bathing frequency (X^2^ = 5521; *p* = 0.3560) and heat-related behaviors such as excessive drinking water (X^2^ = 5550; *p* = 0.1360) and pouring water (X^2^ = 7617; *p* = 0.0550) (Table 2). However, there was an association between owners’ perception about the sensitivity of their dogs to heat and other reported heat-related behaviors, such as placing paws in water (X^2^ = 10,865; *p* = 0.0120), lying on water (X^2^ = 18,370; *p* = 0.0001) and panting (X^2^ = 34,648; *p* = 0.0001) (Table 2).

Regarding the criteria for choosing the time of day to walk the dog (Table 3), “concern about solar radiation” and “time availability” were the most mentioned at all times. Owners who reported “solar radiation” as criteria also were more likely to report walking with their dogs at early morning, late afternoon and nighttime. Overall, 97% of respondents answered that they walked with their dogs during these times. For respondents who reported walks between 10:00 and 16:00, 60% of answers gave “time availability” as the principal criteria for choosing this time.

## 4. Discussion

A widely disseminated notion is that breeds endogenous to a cold region may find it difficult to cope with warm climates [18]. The Husky Siberian is a breed originating from cold temperate climates and that was imported to Brazil, a typical tropical country, where levels of solar irradiance are high and almost constant over the year [19]. This study is the first to assess owners’ perceptions about the heat tolerance of their Husky Siberian dogs, and to see the association with owners’ attitudes towards routine care of their dogs. Our study produced three important findings: First, owners reported several heat-related behaviors expressed by their dogs. Second, most of the respondents perceived their dogs as low-heat-tolerant animals, and this perception seemed to be associated with some heat-related behaviors of the dogs noticed by the owners. Third, this perception was also linked to positive attitudes towards protecting dogs against extreme hot weather conditions, as most of the owners (~97%) reported choosing more thermally comfortable times for walking with their dogs.

### 4.1. Owners’ Perception and Thermoregulatory Behavior Expressed by Their Dogs

Our findings suggest that there was an association between thermoregulatory behaviors and the belief that the dogs are sensitive to heat. Owners reported several heat-related behaviors expressed by their dogs, such as excessive water consumption, shade-seeking, lying on a wetted surface, pouring water, placing paws in water, and panting. Even though they may not fully understand the biophysics or purpose of a specific thermoregulatory response, these behaviors are widely known as voluntary mechanisms that endothermic animals, including humans (with the exception of panting), employ to enhance the transference of body heat to the environment [12]. However, based on the contingency table, while some reported behaviors were associated with owners’ perceptions about the low heat tolerance of their dogs (e.g., placing paws in water fountains, lying in water, and panting), others were not associated., e.g., excessive water consumption and pouring water.

The behavior of placing the paws in water and lying on water facilitate the dissipation of body heat by conduction and evaporation. When compared to other body regions, the lower number of hairs and denser network of blood vessels in the paws, a body region commonly referred to as a thermal window in dogs, help to decrease resistance towards heat and moisture being transferred to the environment [20]. Additionally, placing the paws in a water trough is likely to be a behavior that initiates a sequence of attempting to spill water and subsequently lying on top of it (personal observation). This behavior results in a larger surface area that is wetted, which in turn enhances the evaporative heat loss, a good example of how animals voluntarily manipulate rates of heat and moisture exchanges with their environment.

Exposure to hot environments can lead dogs to increase open-mouth respiration, an autonomic response known as panting or thermal tachypnoea that also enhances heat loss by evaporation from the upper surface of the respiratory tract [21]. The respiratory tract is the principal avenue that dogs use to transfer latent heat to the environment, as they do not possess active sweat glands [22]. This response is easy to detect visually. In this study, most of the owners who perceived their dogs to be low-heat-tolerant animals also reported their dogs panting. During panting, dogs increase minute ventilation by increasing their respiratory rate and decreasing their tidal volume, a type of shallow breathing that normally preserves alveolar gas exchange [23]. However, exposure of dogs to extreme hot conditions, such as during walks at times of high radiant heat load, can turn panting to thermal hyperventilation and increase the susceptibility of dogs to developing respiratory alkalosis and heat stroke [22,24].

### 4.2. Owners’ Perceptions and Attitudes Concerning Routine Care of Their Dogs

Owners seemed to associate heat sensitivity of dogs with the behavior of lying down on water, but they did not make the same connection with bathing, despite it being a practice that can also assist the thermoregulation of dogs. This result indicates that while owners perceive Huskies as low-heat-tolerant animals, certain management practices do not align with this perception. Even in cases where baths were reported to be more frequent, it is possible that this could be related to the dogs’ behavior of spilling water and lying down on it, which could cause them to get dirty more easily, requiring another new bath. Thus, owners seem to perceive baths solely as hygienic and/or aesthetic procedures, without considering their potential as a heat abatement strategy.

**Figure 3:**
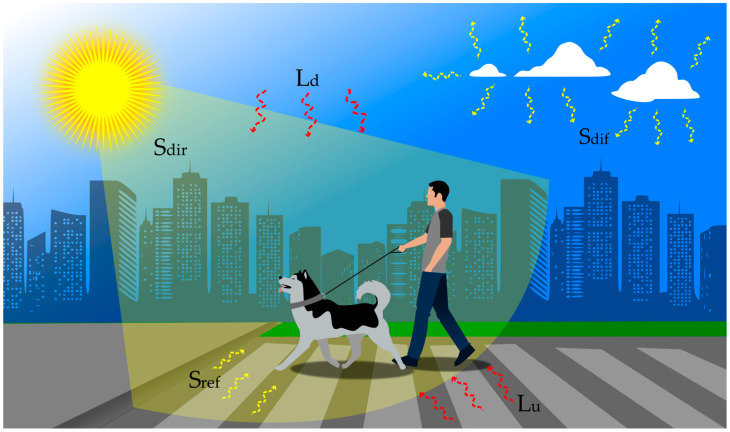
Representation of the principal sources of thermal radiation that dogs and owners are exposed to while walking in a tropical sunny region. S_dir_, S_dif_ and S_ref_ are, respectively, direct, diffuse and reflected short-wave solar radiation; L_d_ and L_u_ are, respectively, downward long-wave radiation emitted from the sky and upward radiation emitted from the ground surface and surrounding objects.

Our results, however, suggest an association between perceptions about the heat sensitivity of dogs and the positive attitude of their owners to avoid walks during times of high radiant heat load. Most of them reported walking with dogs during early morning, late afternoon or nighttime, and also reported solar irradiance as being the principal criteria for choosing these times. However, those owners who reported walking with their dogs during times of high solar input, e.g., between 10:00 and 16:00, mentioned a lack of available time as the principal choosing criteria. In tropical areas, between 10:00 and 16:00, dogs and owners can be exposed to as much as 1200 W m^−2^ of solar irradiance while walking and absorb radiant heat emitted from the sky and surrounding objects (e.g., long-wave radiation), as well as through direct, diffuse and reflected short-wave solar radiation [25] (Figure 3). Dogs, therefore, will face challenges in sustaining their homeothermy when the sum of metabolic heat production and absorbed radiant heat is not offset by the rates of heat transfer to the environment. Under such circumstances, they rely on vigorous panting [9], which in turn increases their susceptibility to developing heat-related illness such as respiratory alkalosis, metabolic acidosis, hypoglycemia and heat stroke [26]. Indeed, the exposure of dogs to prolonged extreme weather conditions has been reported to be the principal risk factor of heatstroke [27]. Other factors such as small body size, dark-colored coat surface, brachycephaly, dehydration and obesity can further increase risks of heatstroke in dogs [26,28,29].

The preference of owners to walk with their dogs at times of less solar input would not only decrease absorbed radiant heat, but would also protect dogs against the negative effects of prolonged exposure to ultraviolet radiation. Short-wave solar radiation, especially the portion within the ultraviolet band (*λ =* 0.200 to 0.390 µm), can be transmitted through the skin and cause serious damage [30]. In tropical areas, the ultraviolet band can account for 10% of the total short-wave solar irradiance [31]. Skin lesions such as solar keratosis or elastosis, solar dermatosis and cutaneous neoplasia have been associated with prolonged exposure of dogs to solar radiation [32]. Body regions with sparse hairs, and nonpigmented skin such as abdomen, eyes and ears, are the areas most susceptible to developing these problems [33,34]. Although Husky dogs possess a well-insulated coat surface, photons of reflected and diffuse solar radiation can be transmitted through the skin in less-haired body regions such as the ventral abdomen and eyes.

The problems derived from prolonged exposure to high levels of solar radiation have been also extensively discussed and disseminated for humans [22,26]. For instance, Ioannou et al. (2021) [27] evidenced that people working while exposed to solar radiation were more susceptible to experience dizziness and weakness, while heart rate, skin blood flow and sweat rate substantially increased and cognitive performance decreased (e.g., attention and vigilance), when compared with people working in shaded conditions. Furthermore, there is robust scientific evidence about the link between exposure to solar radiation and the susceptibility of humans to develop skin cancer [35]. Therefore, whether the preference of owners for choosing more thermally comfortable times to walk with dogs is due to the perception that they are low-heat-tolerant animals or due to their own concerns about the negative impact of solar radiation on their own health is still an unanswered question. By taking into account the one health perspective, the awareness of owners that the thermal environment would impair their own health would also reflect in improvements on dogs’ welfare [36]. Further and more comprehensive studies are, however, warranted concerning the thermal environmental thresholds for dogs living in tropical conditions, with a focus on determining the most appropriate meteorological conditions in which owners should take their dogs for safe and thermally comfortable walks. 

## 5. Conclusions

The findings of this study reinforce that Siberian Husky dogs bred in Brazil exhibit thermoregulatory behaviors that indicate their discomfort with heat, and that these behaviors are recognized by their owners. The majority of respondents’ perceptions of their dogs’ heat sensitivity is reflected in their decision-making process, including opting to walk their dogs during cooler times.

Owners need to be attentive to signs of thermal discomfort in their dogs. It is important to promote educational initiatives to clarify the specific thermoregulatory behaviors of Siberian Huskies. Additionally, seeking professional guidance to develop tailored strategies during hot days, including providing shade, fresh water and limiting intense activities during the hottest hours, is also recommended.

## Figures and Tables

**Table 1:** Questionnaire submitted to owners of Siberian Husky dogs living in Brazil.

Knowledge about the Dog Breed and Dog Management
Do you think your dog is uncomfortable with the heat? a. Yes b. No
2. Walking frequency a. More than once a day b. Once a day c. 1 to 2 times a week d. Occasionally e. I don’t walk my dog
3. Time for a walk with the dog a. 06 am to 10 am b. 10 am to 04 pm c. 04 pm to 06 pm d. 06 pm to 06 am
4. How often do you bathe your dog? a. More than 3 times a week b. 2 to 3 times a week c. Once a week d. Twice a month e. 1 time every 2 months or more
5. What is the criterion for choosing the time for tours? a. The solar radiation b. The air temperature c. Low movement of people and other dogs d. Region security e. I didn’t know how to answer
Perception of thermoregulatory behaviors
6. Do you notice that your dog is uncomfortable with heat at specific times? a. No, I can’t define when b. In the hottest seasons of the year c. In the hottest times of the day d. Always
7. How often do you observe excessive water consumption? a. Very b. Regularly c. Occasionally d. Never e. I do not know how to answer
8. How often do you notice that your dog puts his paws in the water bowl? a. Very b. Regularly c. Occasionally d. Never e. I do not know how to answer
9. How often do you notice that your dog purposely spills the water from the water trough? a. Very b. Regularly c. Occasionally d. Never e. I do not know how to answer
10. How often do you notice your dog lying on water? a. Very b. Regularly c. Occasionally d. Never e. I do not know how to answer
11. How often do you notice your dog panting? a. Very b. Regularly c. Occasionally d. Never e. I do not know how to answer
12. How often does your dog bite taps or hoses? a. Very b. Regularly c. Occasionally d. Never e. I do not know how to answer
13. How often does your dog dig in the dirt to lie down? a. Very b. Regularly c. Occasionally d. Never e. I do not know how to answer
14. How often does your dog look for shade? a. Very b. Regularly c. Occasionally d. Never e. I do not know how to answer
15. How often do you find that your dog tires easily during play? a. Very b. Regularly c. Occasionally d. Never e. I do not know how to answer

**Table 2:** Absolute and relative frequency of bath management and thermoregulatory behaviors in relation to the owners’ perception of their dogs’ sensitivity or not to heat. The chi-square test (or Fisher’s exact test in 2 × 2 tables) shows the associations between the owners’ perception about the heat tolerance of their dogs, owners’ care for their dogs and thermoregulatory behaviors presented by the dogs. Bold “*p*” values are significant.

Routine Care and Behaviors	Total (*n* = 3786)	Heat Sensitivity		X^2^	*p*-Value
Bath Frequency		Yes (*n* = 3162)	No (*n* = 624)		
Three times a week	4 (0.6)	4 (100)	0		
Two/three a week	25 (4)	22 (88)	3 (12)		
Once a week	184 (29.2)	161 (87.5)	23 (12.5)		
Twice a month	151 (23.9)	121 (80.1)	30 (19.9)	5521	0.356
Once a month	226 (35.8)	187 (82.7)	39 (17.3)		
Once every two months	41 (6.5)	32 (78)	9 (22)		
Excessive water consumption					
Much	136 (21.6)	120 (88.2)	16 (11.8)		
Regularly	267 (42.3)	220 (82.4)	47 (17.6)	5550	0.136
Sporadically	147 (23.3)	125 (85)	22 (15)		
Never	81 (12.8)	62 (76.5)	19 (23.5)		
Paws in the water fountain					
Much	195 (30.9)	170 (87.2)	25 (12.8)		
Regularly	121 (19.2)	96 (79.3)	25 (20.7)	10,865	**0.012**
Sporadically	157 (24.9)	139 (88.5)	18 (11.5)		
Never	158 (25)	122 (77.2)	36 (22.8)		
Pouring water					
Much	188 (29.8)	159 (84.6)	29 (15.4)		
Regularly	124 (19.7)	110 (88.7)	14 (11.3)	7617	**0.055**
Sporadically	147 (23.3)	125 (85)	22 (15)		
Never	172 (27.3)	133 (77.3)	39 (22.7)		
Lying on water					
Much	82 (13)	74 (90.2)	8 (9.8)		
Regularly	88 (13.9)	83 (94.3)	5 (5.7)	18,370	**0.000**
Sporadically	136 (21.3)	117 (86)	19 (14)		
Never	325 (51.5)	253 (77.8)	72 (22.2)		
Panting					
Much	103 (16.3)	94 (9.3)	9 (8.7)		
Regularly	214 (33.9)	192 (89.7)	22 (10.3)	34,648	**0.000**
Sporadically	269 (42.6)	215 (79.9)	54 (20.1)		
Never	45 (7.1)	26 (57.8)	19 (42.2)		

**Table 3:** Absolute and relative frequency (within parentheses in %) of the criteria for choosing the times to walk the dogs and walk times. The chi-square test was used to verify the association of these variables.

	Walk Time			
Walking Management	Early Morning (06:01–10:00)	Middle of the Day (10:01–16:00)	Late Afternoon (16:01–18:00)	Nighttime (18:01–06:00)
Criteria for choosing the hours				
Air temperature	16 (9.7)	0	29 (10.3)	29 (8.8)
Solar radiation	109 (66.0)	5 (25.0)	193 (68.4)	197 (60.1)
Movement of people	11 (6.7)	3 (15.0)	9 (3.2)	25 (7.6)
Time availability	26 (15.8)	12 (60.0)	47 (16.7)	77 (23.5)
Region security	03 (1.8)	-	2 (0.7)	-
Did not know how to answer	0	0	2 (0.7)	0
Total (*n* = 795)	165 (20.8)	20 (2.5)	282 (35.5)	328 (41.2)
X^2^	17,088	23,309	34,201	40,442
*p* value	0.004	0.0001	0.0001	0.0001

## Data Availability

Data are available from the corresponding author upon request.

## References

[1] Cook A., Arter J., Jacobs L.F. (2014). My owner, right or wrong: The effect of familiarity on the domestic dog’s behavior in a food-choice task. Anim. Cogn..

[2] Zenithson Y., Bess J.P., Cynthia M.O., Virginia A.B.M., Siracusa C., Stephen R.W. (2014). The effect of dog–human interaction on cortisol and behavior in registered animal-assisted activity dogs. Appl. Anim. Behav. Sci..

[3] Csoltova E., Martineau M., Boissy A., Gilbert C. (2017). Behavioral and physiological reactions in dogs to a veterinary examination: Owner-dog interactions improve canine well-being. Physiol. Behav..

[4] Fukuzawa M., Kajino S. (2018). Auditory Stimuli as Environmental Enrichment Tool for Family Dogs. Int. J. Biol..

[5] Sampaio R.A.G., Martins Y.N.F., Barbosa F.M.S., Franco C.I.Q., Kobayashi M.D., Talieri I.C. (2019). Behavioral assessment of shelter dogs submitted to different methods of environmental enrichment. Ciência Rural.

[6] Amaya V., Paterson M.B.A., Phillips C.J.C. (2020). Effects of Olfactory and Auditory Enrichment on the Behaviour of Shelter Dogs. Animals.

[7] Potter K., Baldwin M.S. (2019). Dogs as Support and Motivation for Physical Activity. Curr. Sports Med. Rep..

[8] Hall E.J., Carter A.J., Farnworth M.J. (2021). Exploring owner perceptions of the impacts of seasonal weather variations on canine activity and potential consequences for human–canine relationships. Animals.

[9] Veríssimo T.N.S. (2021). Heat Tolerance and Welfare of Domestic Dogs Raised in Brazil. Doctoral Thesis.

[10] Nascimento P.V. (2023). Thermoregulation of Domestic Dogs during a Walk in a Tropical Environment. Master’s Dissertation.

[11] Neander C., Baker J., Kelsey K., Feugang J., Perry E. (2021). A comparison of black vs. yellow coat color on rectal and gastrointestinal temperature in Labrador retrievers. J. Vet. Behav..

[12] Maia A.S.C., Silva R.G., Nascimento S.T., Nascimento C.C.N., Pedroza H.P., Domingos H.G.T. (2015). Thermoregulatory responses of goats in hot environments. Int. J. Biometeorol..

[13] Flournoy S.W., Wohl J.S. (2003). Heatstroke in dogs: Pathophysiology and predisposing factors. Compend. Contin. Educ. Vet..

[14] Piacentini R.D., Salum G.M., Fraidenraich N., Tiba C. (2011). Extreme total solar irradiance due to cloud enhancement at sea level of the NE Atlantic coast of Brazil. Renew. Energy.

[15] Protopopova A., Ly L.H., Eagan B.H., Brown K.M. (2021). Climate Change and Companion Animals: Identifying Links and Opportunities for Mitigation and Adaptation Strategies. Integr. Comp. Biol..

[16] Hinkemeyer B., Januszewski N., Julstrom B.A. (2006). An expert system for evaluating Siberian Huskies. Expert Syst. Appl..

[17] Eriksson M., Keeling L.J., Rehn T. (2017). Cats and owners interact more with each other after a longer duration of separation. PLoS ONE.

[18] Berman A. (2011). Invited review: Are adaptations present to support dairy cattle productivity in warm climates?. J. Dairy Sci..

[19] Fonsêca V.F.C., Silva R.G., Moura G.A.B., Snelling E.P., Fuller A., Mitchell D., Costa C.C.M., Milan H.M., Maia A.S.C. (2022). Reliability of methods to determine cutaneous evaporative water loss rate in furred and fleeced mammals. J. Exp. Zool. A Ecol. Integr. Physiol..

[20] Jimenez A.G., Paul K., Zafar A., Ay A. (2023). Effect of different masses, ages, and coats on the thermoregulation of dogs before and after exercise across different seasons. Vet. Res. Commun..

[21] Jennings D.B., Macklin R.D. (1972). The effects of o2 and co2 and of ambient temperature on ventilatory patterns of dogs. Respir. Physiol..

[22] Goldberg M.B., Langman V.A., Taylor C.R. (1981). Panting in dogs: Paths of air flow in response to heat and exercise. Respir. Physiol..

[23] Proscurshim P., Russo A.K., Silva A.C. (1989). Efeitos do treinamento aeróbico no consumo máximo de oxigênio, limiar de lactato e desaparecimento de lactato durante a recuperação do exercício de cães. Bioquímica Comp. E Fisiologia. A Fisiol. Comp..

[24] Baltzer W.I., Firshman A.M., Stang B., Warnock J.J., Gorman E., McKenzie E.C. (2012). The effect of agility exercise on eicosanoid excretion, oxidant status, and plasma lactate in dogs. Vet. Res..

[25] Mitchell D., Snelling E.P., Hetem R.S., Maloney S.K., Strauss W.M., Fuller A. (2018). Revisiting concepts of thermal physiology: Predicting responses of mammals to climate change. J. Anim. Ecol..

[26] Bruchim Y., Klement E., Saragusty J., Finkeilstein E., Kass P., Aroch I. (2006). Heat Stroke in Dogs: A Retrospective Study of 54 Cases (1999–2004) and Analysis of Risk Factors for Death. J. Vet. Intern. Med..

[27] Ioannou L.G., Tsoutsoubi L., Mantzios K., Gkikas G., Pill J.F., Dinas P.C., Notley S.R., Kenny G.P., Nybo L., Flouris A.D. (2021). The Impacts of Sun Exposure on Worker Physiology and Cognition: Multi-Country Evidence and Interventions. Int. J. Environ. Res. Public Health.

[28] Crawford E.C. (1962). Mechanical aspects of panting in dogs. J. Appl. Physiol..

[29] Kanter G.S. (1959). Cause of hypoglycemia in dogs exposed to heat. Am. J. Physiol..

[30] Da Silva R.G., La Scala Jr N., Pocay P.L.B. (2001). Transmission of Ultraviolet Radiation Through the Haircoat and the Skin of Cattle. Rev. Bras. Zootec..

[31] Maia A.S.C., Culhari E.A., Fonsêca V.F.C., Milan H.F.M., Gebremedhin K.G. (2020). Photovoltaic panels as shading resources for livestock. J. Clean. Prod..

[32] Nikula K.J., Benjamin S.A., Angleton G.M., Saunders W.J., Lee A.C. (1992). Ultraviolet Radiation, Solar Dermatosis, and Cutaneous Neoplasia in Beagle Dogs. Radiat. Res..

[33] Madewell B.R., Conroy J.D., Hodkins E.M. (1981). Sunlight-skin cancer association in the dog: A report of three cases. J. Cutan. Pathol..

[34] Hargis A.M., Thomassen R.W., Phemister R.D. (1997). Chronic Dermatosis and cutaneous squamous cell carcinoma in the beagle dog. Vet. Pathol..

[35] Neale R.E., Lucas R.M., Byrne S.N., Hollestein L., Rhodes L.E., Yazar S., Young A.R., Berwick M., Ireland R.A., Olsen C.M. (2023). The efects of exposure to solar radiation on human health. Photochem. Photobiol. Sci..

[36] Pinillos R.G., Appleby M.C., Manteca X., Scott-Park F., Smith C., Velarde A. (2016). One Welfare—A platform for improving human and animal welfare. Vet. Rec..

